# Linking *APOE4/4* genotype to microglial lipid droplets and neurotoxicity in Alzheimer’s disease

**DOI:** 10.1186/s40035-024-00433-w

**Published:** 2024-07-30

**Authors:** Hao Huang, Rong Xiang, Riqiang Yan

**Affiliations:** 1 Department of Cell Biology, School of Life Sciences, Central South University, Changsha, Hunan China; 2 Hunan Key Laboratory of Organ Fibrosis, Central South University, Changsha, Hunan China; 3 Department of Neuroscience, University of Connecticut Health, Farmington, CT USA

Alzheimer’s disease (AD) is characterized by progressive cognitive decline and is a neurodegenerative disorder that primarily affects the elderly population worldwide. The main neuropathological features of AD include the development of two pathological hallmarks: intraneuronal neurofibrillary tangles and extracellular amyloid plaques, which are often surrounded by microglia, reactive astrocytes and dystrophic neurites [1]. Despite extensive research, the etiology and detailed molecular mechanisms of AD remain an intriguing enigma.

In 1907, Alois Alzheimer first described the accumulation of lipids within cells surrounding amyloid plaques as a principal neuropathological feature of AD. However, the molecular mechanism underlying this lipid accumulation was not understood. It was not even clear whether this is a cause or a consequence of the pathology. Early human genetic and recent large-scale genome-wide association studies have identified the apolipoprotein E (*APOE*) ε4 allele as the strongest genetic risk factor for both sporadic and late-onset AD [2]. The prototype function of APOE is a lipid carrier, which primarily facilitates the intercellular transport of cholesterol. In AD brains, the synthesis of APOE is markedly increased, both in astrocytes and microglia. Elevated APOE levels are likely to exert its effects by mediating cholesterol transport or by activating various receptor signaling pathways, although its exact role in AD remains to be clarified.

Microglia are the resident immune cells of the central nervous system. In AD, microglia lose their homeostatic molecular characteristics and undergo a phenotypic transition into disease-associated microglia (DAM) with a more metabolically active state. DAMs are characterized by upregulated expression of genes such as *APOE* and *TREM2*, and the downregulated expression of genes such as *CX3CL1* and *P2RY12*. They also exhibit increased production of cytokines (including IL-18 and IL-1β) and elevated levels of reactive oxygen species (ROS). Recent studies have shown that lipid metabolism dysregulation, manifested as the accumulation of lipid droplets (LDs), occurs in a subset of microglia known as lipid-droplet-accumulating microglia (LDAM) [3]. These cells may represent a distinct disease-associated microglial state or subtype. Unlike DAM, LDAM lack phagocytic function, but secrete pro-inflammatory factors, which may play a crucial role in aging and the development of neurodegenerative diseases.

In a recent *Nature* paper, Haney et al*.* highlight the pivotal role of lipid accumulation in AD pathogenesis [4]. They found that lipid bodies stained with Oil Red O are significantly increased in the brains of AD patients and are commonly located at or near the core of amyloid-beta (Aβ) plaques. Single-nucleus RNA sequencing identified a specific, lipid-associated microglia subtype positive for acyl-CoA synthetase long chain family member 1 (ACSL1), which is most abundant in AD patients with the *APOE4/4* genotype. This subtype highly expresses metabolic and LD-associated genes and is located near Aβ plaques. ASCL1 is a key enzyme with basic helix-loop-helix domain and plays an essential role in lipid biosynthesis including regulation of triglyceride levels through the PPARγ pathway. The number of lipid bodies, which positively correlates with ACSL1 expression by microglia, showed negative correlations with the cognitive performance of patients and positive correlations with the number of Aβ plaques and the level of tau protein pathology. Furthermore, when *APOE4/4* induced pluripotent stem cell-derived microglia (iMG) were stimulated with fibrillar Aβ (fAβ), significant increases in ACSL1 expression and LD accumulation were observed. Lipidomics and coherent anti-Stokes Raman scattering imaging confirmed that the upregulated lipids were primarily triglycerides. The ACSL1 inhibitor Triacin C significantly reversed the accumulation of LDs in *APOE4/4* iMG upon fAβ stimulation.

Mechanistically, ATAC-seq and RNA-seq analyses on LD-high and LD-low iMG revealed that the LD-high iMG exhibited an enrichment of motifs related to the NF-κB family of transcription factors. The LD-high microglia also showed higher expression of NF-κB-associated pro-inflammatory cytokines, such as TNFα and IL1β. Using a CRISPR-KO screening library to analyze genetic modifiers of LD accumulation in *APOE4/4* iMG following fAβ stimulation, the catalytic subunit of phosphoinositide 3 kinase (PI3K), PIK3CA, was identified as the top hit. Furthermore, treatment with the PI3K inhibitor GNE-317 significantly reduced LD accumulation in *APOE4/4* iMG cells stimulated with fAβ and notably reversed lysosome levels and the secretion of inflammatory cytokines. When *APOE4/4* iPSC-derived human neurons were cultured with conditioned media from *APOE4/4* LD-high iMG, authors found phosphorylation of tau protein, activation of caspase-3, and increased lipid accumulation in iPSC-derived neurons. Lipidomic analysis confirmed that the increased lipids were primarily triglycerides. Additionally, conditioned media from both *APOE3/3* and *APOE4/4* iPSC-derived iMG showed similar effects, albeit at varying degrees, while conditioned media from *APOE*-KO iMG did not have these effects (Fig. 1).

**Fig. 1:**
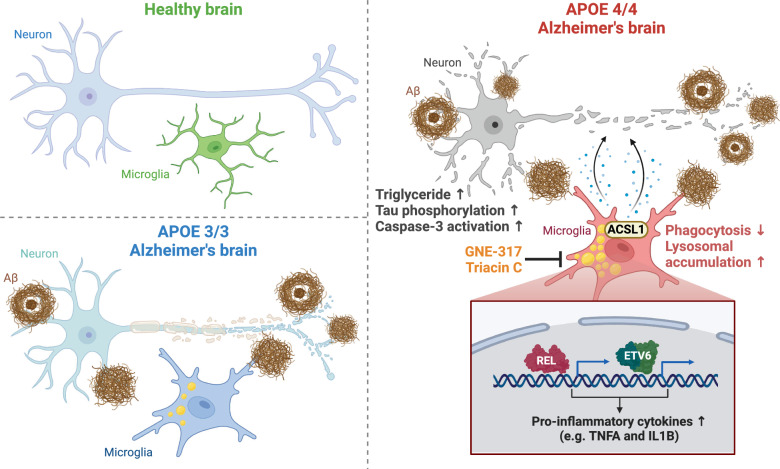
Summary of the impact of microglial lipid droplet (LD) accumulation in AD. Compared to normal controls and *APOE3/3* AD patients, ACSL1^+^ microglia identified in *APOE4/4* AD patients are enriched with triglyceride LDs, which are distributed around the core or the periphery of Aβ plaques. These cells exhibit lysosomal accumulation and reduced phagocytosis. ACSL1 inhibitor Triacin C or a PI3K inhibitor GNE-317 significantly reverses LD accumulation in microglia. LD-accumulating microglia show an enrichment of motifs related to the NF-κB family of transcription factors (such as REL and ETV6) and higher expression of pro-inflammatory cytokines (such as TNFα and IL1β). These factors induce high levels of tau protein phosphorylation, caspase-3 activation, and increased triglyceride accumulation in neurons, thereby contributing to neurodegeneration. Figure created with BioRender.com

Overall, this study elucidates the role of microglial LD accumulation in AD pathology, delving into the role of ACSL1 in LD formation and neurotoxicity in microglia. While the specific molecular mechanisms of LD accumulation and neurotoxicity require further exploration, a study further corroborated Haney’s findings by focusing on mice with deletion of nuclear receptor REV-ERBα in microglia [5]. REV-ERB regulates lipid metabolic, neuronal, and inflammatory functions. They showed that microglial REV-ERBα deficiency causes LD accumulation in microglia and impairs microglial tau phagocytic activity. Indeed, using oleic acid to induce LD accumulation in microglia significantly inhibits the uptake of tau protein. These findings together suggest that LD accumulation, inflammation, and tau uptake may have a synergistic effect.

Consistent with previous research [6], the study by Haney et al. extends prior investigations into the role of the *APOE4* allele in microglial lipid accumulation and its impact on neuron-microglia communication. It further elucidates the specific mechanisms by which ACSL1^+^ LDAM induce neuronal tau phosphorylation and neurotoxicity. Park et al. discovered that microglia-like cells (iMicro) differentiated from human iPSC-derived primitive macrophages, when co-cultured with brain organoids, may transfer cholesterol to neuronal cells via high-density lipoprotein (HDL) containing APOE and lipoprotein lipase. Although the specific mechanism of this transfer remains unclear, they demonstrated that the cholesterol from iMicro can be utilized by neuronal cells and participate in the growth and development of the brain organoids [7]. Conversely, downregulation of AMPK in neurons can promote the accumulation of LDs within the neurons themselves and transfer excess lipids to microglia. This subsequently triggers microglial LD accumulation, inflammatory responses and phagocytic dysfunction, creating a vicious cycle. This illustrates that lipid transfer between neurons and microglia is a mutual process [8].

In AD pathology, neutral lipid accumulation in neurons has also been discovered. By using a neutral lipid fluorescent dye BODIPY 500/510, our group detected co-staining of neutral lipids with neurofilament light, surrounding Aβ plaques in the cortical region of APP^NL-G-F^ mouse brains and AD human samples [9]. We also revealed that reticulon 3, an important tubular endoplasmic reticulum protein that plays a role in lipid metabolism and AD pathological changes [10], may mediate the accumulation of lipids in neuronal dystrophic neurites and contribute to Aβ release [9]. Our findings align with the increased LipidSpot staining in neurons cultured with LD-high iMG conditioned media [4]. Exploring lipid accumulation and its impact on microglia and neurons may advance the understanding of the complex pathological processes of AD.

In summary, the *APOE4/4* genotype causes a lipid metabolic imbalance and has an impact on the development of AD. These insights pave the way for targeted therapies against LD accumulation in microglia during the progression of AD.

## Data Availability

Not applicable.

## Abbreviations

AD: Alzheimer’s disease
Aβ: Amyloid-beta
APOE: Apolipoprotein E
DAM: Disease-associated microglia
ROS: Reactive oxygen species
LDs: Lipid droplets
LDAM: Lipid-droplet-accumulating microglia
fAβ: Fibrillar Aβ
iMG: Induced pluripotent stem cell-derived microglia
